# Effectiveness of the available early therapies in reducing severe COVID-19 in non-hospitalized patients with solid tumors on active treatment

**DOI:** 10.3389/fmed.2022.1036473

**Published:** 2022-10-26

**Authors:** Angioletta Lasagna, Irene Cassaniti, Daniele Lilleri, Mattia Quaccini, Alessandra Ferrari, Paolo Sacchi, Raffaele Bruno, Fausto Baldanti, Paolo Pedrazzoli

**Affiliations:** ^1^Medical Oncology Unit, Fondazione IRCCS Policlinico San Matteo, Pavia, Italy; ^2^Molecular Virology Unit, Department of Microbiology and Virology, Fondazione IRCCS Policlinico San Matteo, Pavia, Italy; ^3^Division of Infectious Diseases I, Fondazione IRCCS Policlinico San Matteo, Pavia, Italy; ^4^Department of Clinical, Surgical, Diagnostic and Pediatric Sciences, University of Pavia, Pavia, Italy; ^5^Department of Internal Medicine and Medical Therapy, University of Pavia, Pavia, Italy

**Keywords:** remdesivir, nirmatrelvir/ritonavir, real-life data, COVID-19, vaccine, cancer

## Abstract

Emergency use authorization of drugs against severe acute respiratory syndrome coronavirus 2 (SARS-CoV-2) by regulatory authorities has provided new options to treat high-risk outpatients with mild-to-moderate Coronavirus disease 2019 (COVID-19). We conducted an ambispective cohort study of patients with solid tumors on active treatment to examine the effectiveness of these drugs in preventing the progression to severe COVID-19. Sixty-nine patients with solid tumors (43 women, 26 men; median age 61, range 26–80) reported a laboratory-confirmed diagnosis of SARS-CoV-2 infection. Forty-nine patients received early therapy. Only one patient (14.5%) required hospitalization for COVID-19. As for safety, two patients (5.9%) reported nausea during nirmatrelvir/ritonavir. The majority of treated patients showed a reduced time to negative sample (73 *vs.* 18%, *p* = 0.0011) and shorter symptoms’ duration (94 *vs.* 27%; *p* < 0.0001) compared to the patients not treated with the early COVID-19 therapies. Our data suggest that early therapies may reduce the morbidity of COVID-19 in patients with solid tumors.

## Introduction

Despite coronavirus disease 2019 (COVID-19) vaccination being effective for patients with cancer (1), a systematic review has reported that the occurrence of breakthrough infections (BTIs) is more common in immunocompromised individuals than in fully vaccinated healthy individuals (2). Emergency use authorization of drugs against severe acute respiratory syndrome coronavirus 2 (SARS-CoV-2) by regulatory authorities has provided new options to treat high-risk outpatients with mild-to-moderate COVID-19. Their clinical effects were seen in unvaccinated patients, and the trials took place before the emergence of the Omicron variant. The real-world effectiveness of monoclonal antibodies and oral antiviral agents in patients with cancer with COVID-19 is largely uncharacterized (3). We conducted an ambispective cohort study of patients with solid tumors on active treatment to examine the effectiveness of these drugs in preventing the progression to severe COVID-19.

## Patients and methods

### Study population

Our study population consisted of all the patients treated for a solid tumor at the department of Medical Oncology of the Fondazione IRCCS Policlinico San Matteo (one of the COVID-19 referral hubs in Northern Italy) with a positive SARS-CoV-2 antigen or polymerase chain reaction test from January to July 2022. We decided this start date to ensure that the majority of the cases in the present study were caused by Omicron (4) in order to reduce confounding selection bias. We excluded the patients already hospitalized at the time of their COVID-19 diagnoses. For each patient, an infectious disease specialist chose the most appropriate drug among sotrovimab, molnupiravir, remdesivir, and ritonavir-boosted nirmatrelvir. For the included patients, we recorded sex, age, type of solid cancer, type of treatment, treatment setting (first- or second-line, maintenance after chemo-radiotherapy, neo/adjuvant setting), date of the onset of the symptoms, type and duration of symptoms, re-infections, type of early therapy, and anamnestic COVID-19 vaccination. The above data were collected from the patients’ medical records at the time of the administration of the early therapy and then after 1 week, after 2 weeks, and after 4 weeks.

### Outcomes

The primary endpoint was the evaluation of the rate of hospitalization for COVID-19 disease within 30 days in the patients with cancer using COVID-19 early therapies as per indication. The secondary endpoints were the evaluation of the mortality rate for COVID-19 disease, the time to COVID-19 symptoms resolution, and the safety.

### Statistical analysis

Demographic and clinical data were summarized with descriptive statistics and showed as frequencies and percentages. Median, range, and interquartile range (*IQR*) were provided for continuous variables. Comparisons among categorical variables were performed by Fisher’s exact test, while comparisons among continuous variables were performed using the Mann–Whitney test. Spearman’s test was used for correlations. *P*-values lower than 0.05 were considered significant. All the analyses were performed using GraphPad 8.3.0 (GraphPad, La Jolla, CA, USA).

### Ethical considerations

The study (Co-Ther) was approved by the local Ethics Committee (Comitato Etico Area Pavia) and Institutional Review Board (P-0039959/22). All the subjects had signed informed written consent before enrollment.

## Results

COVID-19-related hospitalization within 30 days from symptom onset and safety were evaluated. Sixty-nine patients with solid tumors (43 women, 26 men; median age 61, range 26–80) reported a laboratory-confirmed diagnosis of SARS-CoV-2 infection when the Omicron variant was predominant. None of the patients had a history of prior SARS-CoV-2 infection. Forty-nine patients received an early therapy: Thirty-four patients (69.4%) received nirmatrelvir/ritonavir, seven patients (14.3%) received remdesivir, while five patients (10.2%) and three patients (6.1%) received molnupinavir and sotrovimab, respectively. Among those who were not treated, five patients refused the therapy, six patients had symptoms for > 5 days by the time they were evaluated, and nine patients were asymptomatic. Breast cancer (33.3%) was the most common tumor subtype. Forty-one patients were on chemotherapy (59.4%), 17 patients (24.6%) were on immunotherapy, and 11 patients were on hormonal therapy (16%) (Table 1). The most common immune checkpoint inhibitor (ICI) was pembrolizumab (eight patients, 47.1%).

**TABLE 1:** Demographic and clinical characteristics.

Variable	Whole sample (*n* = 69)	No treatment (*n* = 20)	Sotrovimab (*n* = 3)	Paxlovid (*n* = 34)	Molnupinavir (*n* = 7)	Remdesivir (*n* = 5)
**Sex, *n* (%)**						
Females	43 (62.3%)	11 (55%)	2 (66.7%)	24 (70.6%)	2 (28.6%)	4 (80%)
Males	26 (37.7%)	9 (45%)	1 (33.3%)	10 (29.4%)	5 (71.4%)	1 (20%)
**Type of tumor, *n* (%)**						
Lung	17 (24.6%)	7 (35%)	0	7 (20.6%)	2 (28.6%)	1 (20%)
Melanoma	4 (5.8%)	1 (5%)	0	3 (8.8%)	0	0
Breast	23 (33.3%)	7 (35%)	1 (33.3%)	12 (35.3%)	1 (14.3%)	2 (40%)
Kidney	1 (1.4%)	0	0	1 (2.9%)	0	0
Gastrointestinal	19 (27.5%)	4 (15%)	2 (66.7%)	9 (26.5%)	4 (57.1%)	1 (20%)
Other	5 (7.4%)	2 (10%)	0	2 (5.9%)	0	1 (20%)
**Type of oncological treatment**						
ICIs	17 (24.6%)	3 (15%)	1 (33.3%)	11 (32.3%)	2 (28.6%)	0
Chemotherapy	41 (59.4%)	14 (70%)	2 (66.7%)	17 (50%)	3 (42.8%)	5 (100%)
Target therapy/ormonotherapy	11 (16%)	3 (15%)	0	6 (17.7%)	2 (28.6%)	0
**Vaccination doses, *n* (%)**						
0	5 (7.2%)	1 (5%)	1 (33.3%)	0	2 (28.6%)	1 (20%)
1	1 (1.5%)	1 (5%)	0	0	0	0
2	4 (5.8%)	1 (5%)	0	3 (8.8%)	0	0
3	57 (82.6%)	16 (80%)	2 (66.7%)	30 (88.2%)	5 (71.4%)	4 (80%)
4	2 (2.9%)	1 (5%)	0	1 (3%)	0	0
**Symptoms on the onset *n* (%)**						
Asymptomatic	9 (13%)	9 (45%)	0	0	0	0
Fever	36 (52.2%)	7 (35%)	2 (66.7%)	18 (52.9%)	6 (85.7%)	3 (60%)
Cough	39 (56.5%)	8 (40%)	2 (66.7%)	20 (58.8%)	5 (71.4%)	4 (80%)
Rinorrea	35 (50.7%)	7 (35%)	2 (66.7%)	21 (61.8%)	2 (28.6%)	3 (60%)
Diarrhea	24 (34.8%)	6 (30%)	1 (33.3%)	14 (41.2%)	1 (14.35)	2 (40%)
Hospital admission or COVID-19 worsening, *n* (%)	1 (1.4%)	0	0	0	1 (14.3%)	0

ICIs, immune-checkpoints inhibitors.

The majority of the patients were vaccinated with three doses of mRNA vaccines (57/69; 89.1%) and the median time between the last vaccination dose and the onset of the symptoms was 7 months (range 1–15). The most common symptom of COVID-19 disease at the initial presentation was cough (56.5%). Only one patient (14.5%) required hospitalization for COVID-19. As for safety, two patients (5.9%) reported nausea during nirmatrelvir/ritonavir. The adverse event was considered as mild in severity in both patients, and nirmatrelvir/ritonavir was continued. COVID-19 “rebound” associated with antiviral drugs is described (5). In our cohort, nobody experienced this phenomenon.

It is well known that there is a high risk for drug–drug interactions (DDI) in polymedicated subjects during treatment with nirmatrelvir/ritonavir, primarily due to the ritonavir component (6, 7). In our cohort, according to the international recommendations (6), nobody experienced DDI.

**FIGURE 1:**
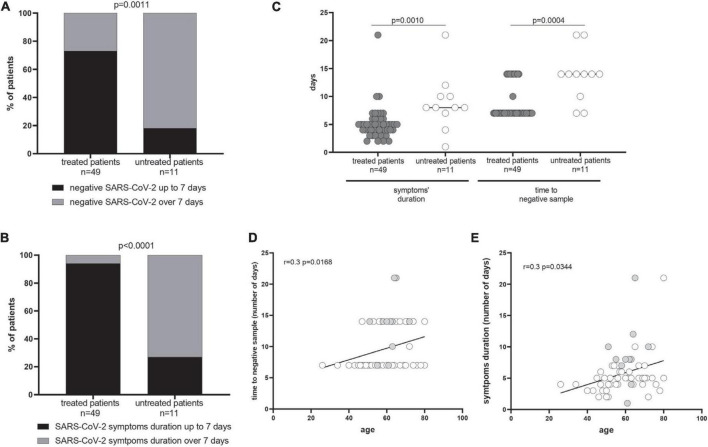
Time to negative severe acute respiratory syndrome coronavirus 2 (SARS-CoV-2) respiratory sample and symptoms’ duration. The percentage of treated and untreated patients with negative SARS-CoV-2 by 7 days and the symptoms’ duration up to 7 days from the symptoms onset are showed in **(A,B)**, respectively. The median time to negative sample in treated patients was 7 days [interquartile range (*IQR*) 7–12)] while it reached 14 days (*IQR* 10–14) in the control group (*p* = 0.0004). The *p*-values were calculated using Fisher’s exact test and given in each graph. In **(C)**, the median number of days of positive SARS-CoV-2 RNA and symptoms’ duration have been reported in treated and untreated patients, and *p-*values were measured using the Mann–Whitney test. A correlation between the age and the symptoms’ duration **(D)** as well as the age and the number of days of positive SARS-CoV-2 RNA **(E)** was measured using the Spearman test, showing separately treated (gray dots) and untreated patients (white dots).

The time to negative SARS-CoV-2 respiratory sample and the symptoms’ duration were compared in 49 early-treated COVID-19 patients and a cohort of 11 symptomatic COVID-19 untreated patients, as controls. The majority of the treated patients showed a reduced time to negative sample (73 *vs.* 18%, *p* = 0.0011) and shorter symptoms’ duration (94 *vs.* 27%; *p* < 0.0001) (Figures 1A,B). The median time to negative sample in treated patients was 7 days (*IQR* 7–12), while it reached 14 days (*IQR* 10–14) in the control group (*p* = 0.0004). The symptoms’ duration was significantly lower in treated than in untreated patients [5 days (*IQR* 4–6) *vs.* 8 days (*IQR* 7–10); *p* = 0.0010] (Figure 1C). A correlation between the number of days for the SARS-CoV-2 negative sample and the age of patients (*r* = 0.3; *p* = 0.0168) as well as between the symptoms’ duration and the age of patients (*r* = 0.3; *p* = 0.0344) was observed (Figures 1D,E). Clinical variables such as the type of therapy (chemotherapy *vs.* immune or target therapy) and the stage of tumor (II–III *vs.* IV) were not associated to the analyzed endpoints.

## Discussion

This study regards the evaluation of the real-world experience with available outpatient COVID-19 therapies in the patients with solid tumors on active treatment. Timely real-world data are important to evaluate the effectiveness and safety of these drugs in frail populations. Outpatient therapies for mild-to-moderate COVID-19 disease have reduced hospitalizations and deaths in clinical trials in unvaccinated subjects before the Omicron surge (8, 9).

We evaluated the effectiveness in terms of the reduction of the COVID-related hospitalization, the duration of symptoms, and the swab negativity in relation to the tumor type, the cancer therapy, and the vaccination status of the patients during the Omicron surge. Our data suggest that the early therapies may reduce the morbidity of COVID-19 in the patients with solid tumors. Only one patient (14.5%) required hospitalization for COVID-19. Moreover, the symptoms’ duration was significantly lower in treated than in untreated patients.

Data on the impact of early therapies come from clinical trials where the groups of patients are pulled together and the subgroup of patients with solid cancers is not analyzed separately. A recent study assessed the effectiveness of remdesivir both in the general population and in 200 patients with hemato-oncological diseases (10), with a significant survival benefit observed at 14 and 30 days for the patients treated with remdesivir.

Our study confirms the validity of the available outpatient COVID-19 therapies in patients with solid tumors on active treatment from a real-world experience. Therefore, oncologists should be aware of the need for the early management of their patients and advise early therapies; in-depth pharmacological anamnesis is also mandatory in the choice of the drug in order to avoid DDI if nirmatrelvir/ritonavir is chosen. We, therefore, recommend a multidisciplinary patient management with close collaboration between the oncologist and the infectious disease specialist.

A limitation of our study is the small sample size due to the monocenter study design. However, this is one of the first papers that specifically assessed cancer patients on active treatment. Future well-powered real-world studies are needed to confirm our results.

## Data availability statement

The raw data supporting the conclusions of this article will be made available by the authors, without undue reservation.

## Ethics statement

The studies involving human participants were reviewed and approved by the Local Ethics Committee (Comitato Etico Area Pavia) and Institutional Review Board (P-0039959/22). The patients/participants provided their written informed consent to participate in this study.

## Author contributions

All authors listed have made a substantial, direct, and intellectual contribution to the work, and approved it for publication.
